# Optimizing Territorial Healthcare Networks with a Capacity-Constrained Hub-And-Spoke Allocation Algorithm: The Province of L’Aquila Case Study

**DOI:** 10.3390/healthcare14070915

**Published:** 2026-04-01

**Authors:** Edoardo Trebbi, Tommaso Barlattani, Antony Bologna, Livia Tognaccini, Alessandro Sili, Giuseppe Di Martino, Cristinel Stan, Camillo Odio, Tommaso Staniscia, Francesca Pacitti, Ferdinando Romano

**Affiliations:** 1 Department of Public Health and Infectious Diseases, “La Sapienza” University of Rome, 00100 Rome, Italy; 2 Department of Biotechnological and Applied Clinical Sciences (DISCAB), University of L’Aquila, Via Vetoio (Coppito 2), 67100 L’Aquila, Italy; 3 Nursing Department, University Hospital Tor Vergata, 00133 Rome, Italy; 4 Department of Medicine and Ageing Sciences, “G. d’Annunzio” University of Chieti-Pescara, 66100 Chieti, Italy; 5 Local Health Authority ASL 1 Avezzano-Sulmona-L’Aquila, 67100 L’Aquila, Italy; cstan@asl1abruzzo.it; 6 Department of Health, Information Flows and Digital Health Service, Abruzzo Region, 65121 Pescara, Italy

**Keywords:** capacity constraints, community health services, hub-and-spoke model, Italy, location allocation, Ministerial Decree 77/2022, spatial equity, territorial health planning, travel-time accessibility

## Abstract

**Background**: Geographic and demographic disparities strongly influence access to community-based healthcare, especially in rural and mountainous areas. In Italy, Ministerial Decree 77/2022 promotes a territorial reorganization based on networked care models, but practical tools for translating policy standards into operational catchment areas remain limited. **Methods**: We developed a transparent, data-driven allocation framework combining travel-time accessibility and population-based capacity constraints. A case study was conducted in the Province of L’Aquila, within Local Health Authority ASL 1 Avezzano–Sulmona–L’Aquila, a low-density mountainous area including 65 municipalities. Using official ISTAT data, including the 2021 national origin–destination road travel-time matrix, municipalities were allocated to 3 hub nodes and 8 spoke nodes. Population caps of 50,000 residents per hub and 40,000 per spoke were applied. Scenario analyses were performed under 20, 30, and 40 min travel-time thresholds. **Results**: Under the 30 min scenario, all municipalities were allocated, but the L’Aquila hub exceeded the capacity cap. A cap-compliant 30 min allocation eliminated this violation at the cost of longer upper-tail travel times. Under the 20 min scenario, only 54 municipalities were allocated, leaving 11 mountainous municipalities outside the threshold. Under the 40 min scenario, all municipalities were allocated without capacity violations. **Conclusions**: The proposed framework provides a reproducible approach for territorial healthcare planning and makes explicit the trade-off between accessibility and capacity compliance in hub-and-spoke network design, particularly in geographically complex mountain settings.

## 1. Introduction

Across many countries, health systems are struggling to keep pace with demographic transitions, the growing burden of chronic disease, and persistent inequalities in access to care [1,2]. Italy is no exception. Regional decentralization has often widened disparities in the availability of essential services, leaving rural and mountainous territories at a particular disadvantage. Recent studies show that residents in these areas face longer travel times to reach healthcare facilities and experience more fragmented care pathways [3,4,5,6]. However, despite the extensive literature on healthcare accessibility, comparatively fewer studies have translated the principles of equity and efficiency into a reproducible, data-driven planning model.

In Italy, increasing life expectancy and the expansion of the older population have led to a sharp rise in chronic conditions such as cardiovascular, respiratory, and metabolic diseases. Managing these conditions requires continuous and coordinated care rather than episodic acute interventions. This demographic shift has intensified the demand for community-based services, adding pressure to a territorial system that already shows signs of fragmentation [7,8].

At the same time, internal migration and demographic decline in inland areas have deepened territorial inequalities. In Italy, provinces such as L’Aquila, characterized by a dispersed settlement pattern across a vast mountainous landscape, exemplify how geography can become a structural barrier to access. In these areas, residents frequently encounter lengthy travel times and limited access to essential health services [4,5]. The province thus represents an exemplary context to test innovative planning tools, as it combines demographic fragility, complex topography, and institutional decentralization. Local Health Districts, historically conceived as the backbone of territorial healthcare, have evolved unevenly across the country, resulting in differences in resources, competencies, and responsiveness to population needs.

The COVID-19 pandemic brought these weaknesses into sharp focus. In Italy, territorial medicine proved unable to absorb the initial shock of the crisis, especially in regions with complex geography, such as Abruzzo, where early triage, home-based management, and contact tracing were particularly difficult. These challenges underscore the urgency of strengthening local health networks and redesigning proximity services. In response, the National Recovery and Resilience Plan (PNRR) and Ministerial Decree 77/2022 [9] introduced a new framework for community-based healthcare organized around Community Health Centers (Case della Comunità), Community Hospitals, and Territorial Operations Centers. The reform seeks to promote equity and ensure uniform Essential Levels of Care (LEA), but its implementation depends on addressing workforce shortages, governance asymmetries, and digital divides [3,10,11]. Evidence from rural and low-resource settings shows that proximity-based systems are most effective when combined with community outreach, mobile units, and telehealth programs [12,13,14].

Within this framework, the hub-and-spoke model has been adopted as the reference architecture for territorial care. Ministerial Decree 77/2022 [10,15] sets out the structural and staffing standards for Community Health Centers, Community Hospitals, and Territorial Operations Centers. The decree extends a network logic already tested in hospital-based systems for stroke, trauma, and oncology, but its application to primary and community care represents an important innovation. The aim is to ensure uniform LEA across all regions, reduce the historical north–south gap, and create resilient networks capable of delivering prevention, chronic disease management, and social health integration close to where people live [3,10,16].

Evidence from Italy and other countries supports the potential of this approach. In Emilia–Romagna, community hospitals have improved coordination between primary and specialist care, although results vary according to local resources and leadership [17]. Pediatric and telestroke networks have demonstrated that combining a hub-and-spoke structure with telemedicine can reduce treatment delays and enhance access equity, particularly in underserved areas [18,19,20]. Systematic reviews of provider-to-provider telehealth and recent discussions on “Hub and Spoke 2.0” highlight the value of digital tools, multidisciplinary collaboration, and two-way communication between hubs and spokes [21,22].

Travel-time accessibility has also become a key metric in assessing health equity. Global high-resolution models suggest that long travel times remain a significant barrier to timely care [23]. Italian data confirm similar disparities, with accessibility gaps particularly pronounced between northern and southern regions, as well as between urban and rural areas [4,7]. Recent studies have developed composite indicators to identify so-called “medical deserts,” combining measures of travel time, service density, and telehealth availability—as shown in Finland, where approximately 13% of the population lives in underserved areas [24]. Comparable findings in Spain link rural depopulation and population aging to increased travel times and reduced hospital access over time [25]. Research on network optimization confirms that data-driven allocation of healthcare resources can strengthen resilience and improve spatial equity in service delivery [26,27]. Building on this literature, the present study moves from accessibility measurement to operational territorial allocation by embedding travel-time logic within a predefined hub-and-spoke architecture and explicit population-cap constraints. This study develops and tests a transparent, capacity-constrained allocation framework to operationalize hub-and-spoke territorial planning using network travel times. Unlike conventional location-allocation or gravity-based accessibility models, the proposed framework is designed to generate directly auditable, policy-oriented outputs under predefined hub-and-spoke nodes and explicit population-cap constraints. Focusing on the Province of L’Aquila (Local Health Authority (ASL) 1 Avezzano–Sulmona–L’Aquila), we quantify travel-time accessibility under alternative planning benchmarks (20, 30, and 40 min), assess the trade-off between proximity-first allocations and compliance with population-based capacity constraints, and provide reproducible outputs that can support local implementation and monitoring of territorial care reforms. Although the case study is local, the method is designed to be portable to other low-density or mountainous settings where spatial equity is a key planning objective. More specifically, the study addresses two main questions: whether full territorial allocation can be achieved under realistic travel-time and capacity constraints, and how the balance between proximity and workload distribution changes across alternative planning scenarios.

## 2. Methods

The study was conducted in the Province of L’Aquila, within the Local Health Authority (ASL) 1, which encompasses Avezzano, Sulmona, and L’Aquila. This territory spans approximately 5048 km^2^ and comprises 65 municipalities, many of which are situated in mountainous or high-altitude regions. Its geography, characterized by scattered settlements, an aging population, and continuous outmigration of younger residents, makes it a suitable setting for testing the accessibility and workload distribution of community health facilities within the hub-and-spoke framework established by Ministerial Decree 77/2022 [3,9,10]. Methodologically, the study combines policy and documentary analysis, geospatial accessibility assessment, heuristic allocation modeling, and comparative scenario evaluation. This integrated approach supports the interpretation of the regulatory framework, the construction of the allocation procedure, and the comparison of alternative planning benchmarks.

Population, land area, and geographic coordinate data were obtained from the Italian National Institute of Statistics (ISTAT). Healthcare facilities were identified and classified as Community Health Centers (Case della Comunità), Community Hospitals (Ospedali di Comunità), and Territorial Operations Centers (Centrali Operative Territoriali), according to the definitions provided by DM 77/2022 and the AGENAS Guidelines for the Implementation of the Hub-and-Spoke Model [9].

Data preparation combined several official ISTAT sources, including the national origin–destination (OD) matrix of road travel times and distances between Italian municipalities (reference year: 2021). The OD matrix provides impedance-adjusted estimates derived from the national road network and reflects realistic travel conditions rather than idealized Euclidean distances. According to ISTAT technical documentation, municipal reference points correspond to the census-section centroid containing the town hall, and OD travel times may be direction-dependent (i.e., not necessarily symmetric). Demographic information disaggregated by age and sex and territorial data on municipal surface area and administrative boundaries were harmonized and merged into a single analytical data frame for the Province of L’Aquila, ensuring consistency across municipal codes and names. Because the OD matrix refers to 2021, potential post-pandemic variations in traffic patterns or infrastructure developments were not incorporated but could be addressed in future updates of the model. Comparable approaches to evaluating spatial accessibility have been widely applied in public health geography, particularly through two-step floating-catchment and gravity-based models [4,28,29].

Candidate hub-and-spoke nodes were defined a priori to reflect the ASL 1 territorial organization and the planned/identified municipalities hosting the main community-care nodes in the study area. We selected three hub nodes (Avezzano, L’Aquila, and Sulmona) and eight spoke nodes (Carsoli, Castel di Sangro, Castelvecchio Subequo, Civitella Roveto, Montereale, Rocca di Mezzo, San Demetrio ne’ Vestini, and Trasacco).

To operationalize workload constraints, we used population-based caps as a proxy for service capacity. DM 77/2022 defines a programming standard of one Casa della Comunità hub per 40,000 to 50,000 residents; we operationalized this as a hub catchment cap of 50,000 residents. For Casa della Comunità spoke, DM 77/2022 indicates that their number and distribution should account for local orographic and demographic characteristics; therefore, we introduced an operational spoke cap of 40,000 residents to prevent spoke centers from functioning as de facto hubs and to preserve a meaningful distinction between hub-and-spoke roles within the present planning framework. These caps should be interpreted as operational planning parameters that may be recalibrated when local staffing and service capacity data are available, rather than as a full representation of facility-level supply or a direct measure of facility-level resources [9]. Within the present framework, lower capacity caps would tend to improve workload balance at the cost of more frequent reallocation, whereas higher caps would preserve proximity-based assignments but increase the risk of concentration at major hubs.

Municipalities were assigned to hub-and-spoke nodes through a multi-phase heuristic algorithm implemented in Python 3. For each municipality, we computed travel times to all candidate nodes and identified a short list of near-optimal candidates within 5% of the municipality’s minimum travel time to reduce instability due to small differences in modeled travel times. Municipalities were prioritized by (i) the number of feasible candidate nodes, in ascending order, (ii) minimum travel time, in ascending order, and (iii) population size, in descending order, so that municipalities with fewer options were allocated first. When ties occurred after the application of these criteria, a fixed ordering rule was applied to preserve reproducibility under identical inputs.

Allocation followed a proximity-first logic, subject to population caps. Each municipality was first considered for assignment to the preferred feasible candidate in its near-optimal set. When assigning a municipality would exceed the capacity of the preferred node, the algorithm activated an iterative reallocation procedure that moved selected municipalities to the next-best feasible alternative in order to reduce constraint violations while minimizing additional travel time. The reallocation cycle continued until capacity violations were resolved or no further feasible reassignment could be identified. Under fixed inputs and ordering rules, the resulting allocation was reproducible. This procedure produced two policy-relevant outputs: (a) a baseline proximity-first allocation that reveals where capacity overloads would occur, and (b) a cap-compliant allocation that resolves overloads at the cost of longer tail travel times.

Scenario analyses were performed using travel-time cut-offs of 20, 30, and 40 min. These thresholds were selected to compare a more restrictive benchmark, an intermediate scenario, and a more permissive scenario in a geographically complex mountainous area. DM 77/2022 does not specify a single universal travel-time standard for territorial access; the selected thresholds were therefore used as planning benchmarks to compare alternative accessibility-capacity scenarios in the study area. For each scenario, we report feasibility (whether municipalities can be allocated under the scenario assumptions), the distribution of travel times (mean, 90th percentile, and maximum), and constraint violations.

**Figure 1:**
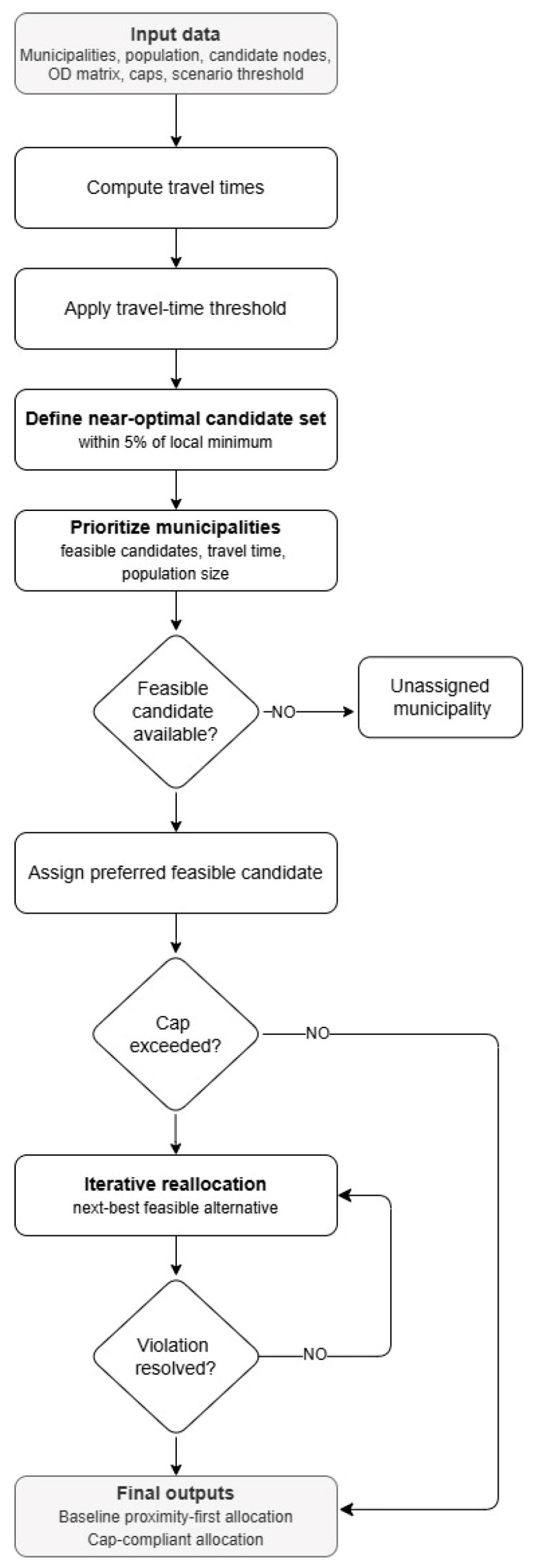
Visual overview of the heuristic allocation workflow. The figure summarizes the main decision steps of the hub-and-spoke assignment procedure, from candidate filtering and municipality prioritization to cap-compliant reallocation. A step-wise pseudocode description is reported in Algorithm S1 in the Supplementary Material.

All computations were performed in Python 3 [30] using the Pandas 2 [31] and OpenPyXL 3 [32] libraries. Standard Python data structures such as heapq, sort, defaultdict, and set were employed to manage prioritization and ensure the unique assignment of each municipality. Computational burden was modest in the present case study, given the limited number of municipalities and candidate centers. The optimization logic was heuristic rather than exact, prioritizing transparent and reproducible allocation under explicit travel-time and capacity constraints over formal global optimization. The algorithm generated an Excel dataset that summarized the final configuration of assignments, the cumulative population served by each center, and overall network indicators. The main decision steps of the workflow are summarized in Figure 1, and a step-wise pseudocode description of the allocation procedure is provided in Algorithm S1 in the Supplementary Material.

## 3. Results

In the 30 min scenario, all 65 municipalities of the Province of L’Aquila were allocated to the three hub nodes (L’Aquila, Avezzano, and Sulmona) and the associated spoke nodes. Travel times were generally short (mean 17 min; 90th percentile 28 min), with a maximum observed travel time of 32 min, reflecting the geographic constraints of peripheral and high-altitude municipalities.

**Figure 2:**
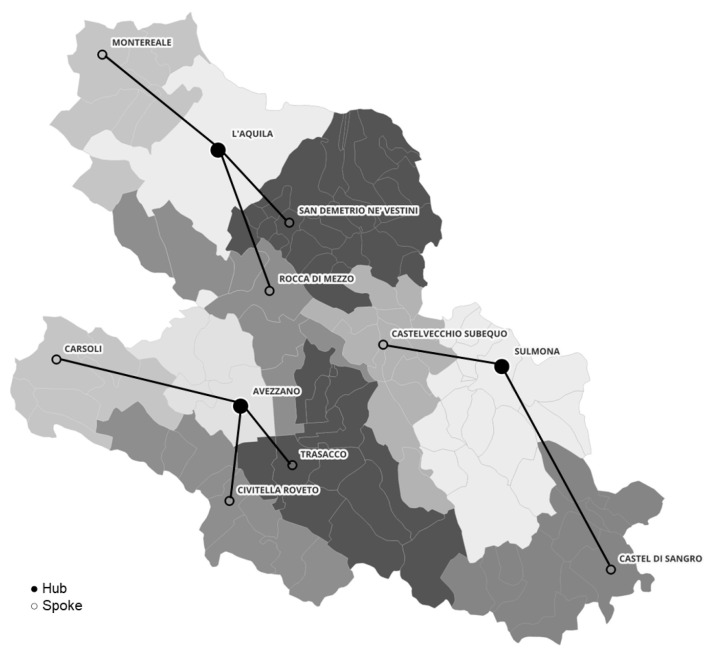
Hub-and-spoke configuration and municipal catchment areas in ASL 1 Avezzano–Sulmona–L’Aquila. The map illustrates the overall spatial structure of the network, showing the municipalities assigned to each center and the links between connected nodes. Different shades of gray identify municipal catchment areas, while connecting lines represent hub–spoke relationships within the service network.

Population distribution across the network revealed a marked imbalance: the L’Aquila hub reached 69,717 residents, exceeding the 50,000-inhabitant limit defined by DM 77/2022, whereas the Avezzano and Sulmona hubs remained within the standard, serving 48,319 and 40,334 residents, respectively. In the baseline 30 min allocation, the L’Aquila hub therefore exceeded the 50,000-resident cap by 19,717 residents, highlighting the tension between proximity-based assignment and capacity compliance. Table 1 summarizes the population served and mean travel times across hubs and spokes, providing an overview of territorial balance under the baseline configuration. Figure 2 illustrates the geographic configuration of the hub-and-spoke network and municipal catchment areas and network configuration across ASL 1, Avezzano–Sulmona–L’Aquila.

Average travel times ranged from 14 to 24 min across the network, with most municipalities meeting the 30 min benchmark. Overall, 90% of municipalities were located within 30 min of their assigned center, and approximately half within 20 min. The highest concentration of short travel times was observed in the Avezzano area, whereas peripheral and high-altitude municipalities approached the upper end of the threshold range. More specifically, mean travel times were 14 min for the Avezzano hub, 19 min for the L’Aquila hub, and 19 min for the Sulmona hub, while among spoke nodes, the highest mean value was observed at Castel di Sangro (24 min) and the lowest at Civitella Roveto (14 min). This pattern was also reflected in isolated inland cases, such as Opi assigned to the Castel di Sangro spoke, where travel time reached 50 min. Spatial analysis revealed clear geographic patterns of accessibility, with shorter travel times concentrated in the Marsica basin, while higher values were observed in the Alto Sangro and Sirente–Velino areas. A comparison among the three hub territories revealed similar average travel times but substantial differences in population density. The Avezzano area displayed the highest density and the most compact service coverage, whereas the L’Aquila and Sulmona areas included several sparsely populated, mountainous municipalities. These spatial variations highlight the enduring impact of geography and infrastructure on service accessibility, even within a standardized network architecture. From a territorial planning perspective, this pattern indicates that a formally uniform network may still yield uneven practical accessibility, making geographic morphology and settlement dispersion crucial factors in calibrating hub-and-spoke coverage.

A cap-compliant variant of the model was developed to correct the overload observed at the L’Aquila hub by reallocating selected municipalities to Avezzano and Sulmona. This adjustment reduced the population served by L’Aquila below the 50,000-resident threshold but slightly increased overall travel times, with the 90th percentile rising from 28 to 34 min and the maximum travel time increasing from 32 to 38 min. This trade-off highlights the intrinsic tension between compliance with regulatory capacity limits and the objective of maintaining equitable accessibility. The adjustment, therefore, converted a proximity-optimal but overloaded configuration into a capacity-compliant one, with only a modest increase in tail travel times.

Scenario analysis highlighted how feasibility and performance depend on the travel-time benchmark (Table 2). Under the strictest 20 min cut-off, only 54 municipalities (88% of the provincial population) could be allocated without exceeding the nominal benchmark, leaving 11 mountainous municipalities outside the standard. At 30 min, allocations were feasible for all municipalities, but the proximity-first solution produced a capacity overload at the L’Aquila hub. The cap-compliant 30 min variant resolved this violation through reallocation, at the cost of higher tail travel times (90th percentile, 34 min; maximum, 38 min). At 40 min, allocations were feasible without capacity violations, with a maximum travel time of 36 min.

Overall, these results show that a travel-time-based, capacity-constrained allocation can effectively reproduce the logic of the hub-and-spoke model under the complex conditions typical of mountain territories while highlighting the trade-off between stricter travel-time thresholds and full territorial coverage.

## 4. Discussion

This study demonstrates that a spatially explicit hub-and-spoke allocation can achieve nearly universal coverage around a 30 min travel-time benchmark in a mountainous and low-density region, consistent with recent Italian reforms in territorial healthcare planning [10,33]. Taken together, the findings indicate that full territorial allocation is achievable under realistic planning thresholds, but the balance between proximity and workload distribution depends strongly on the adopted threshold and on the need to correct hub overload. In its baseline configuration, the model achieved full coverage but revealed an imbalance at the L’Aquila hub, which exceeded its designated capacity. Adjusting population caps ex ante corrected this overload but increased high-percentile and maximum travel times, underscoring the unavoidable tension between equitable accessibility and adherence to planning standards [4,7]. The comparison across scenarios showed that performance depends strongly on the chosen threshold: while a 20 min cut-off excluded several mountain municipalities, a 40 min limit guaranteed universal coverage but compromised timeliness. Taken together, these findings suggest that the framework is both resilient and adaptable to the province’s complex geography.

At the heart of this work is a heuristic, multi-phase allocation algorithm that translates the principles of Ministerial Decree 77/2022 and the AGENAS guidelines for Community Health Centers into operational terms [34]. Unlike traditional cartographic approaches to accessibility [35,36], the algorithm integrates network-based travel time with capacity constraints and a swap mechanism that redistributes demand while minimizing increases in travel time, producing outputs directly interpretable by planners. This feature enhances transparency and facilitates the translation of technical findings into policy decisions. Building on established work in spatial optimization [37,38,39], this framework aligns methodological rigor with current health-policy objectives. Because it relies solely on official statistics (ISTAT data, municipal demographics) and open-source tools, it can be easily adapted to other regions with similar geographic and institutional profiles.

Adopting a 30 min benchmark represents a pragmatic compromise between clinical relevance and territorial feasibility, consistent with international accessibility research that treats travel time as a proxy for equity in access to care [23]. In emergency medicine, even modest increases in travel time have been linked to worse outcomes [40], and high-resolution spatial analyses show that populations living beyond roughly half an hour from care experience greater vulnerability and less continuity of service [8,41]. Within Italy, the experience of community hospitals confirms that proximity fosters integration, although results depend heavily on local resources and governance [17]. Similarly, pediatric and emergency networks demonstrate that structured hub-to-spoke coordination, often supported by telehealth, can reduce delays and improve equity [13,18,19,21]. From a planning perspective, the three thresholds should not be interpreted as interchangeable technical options but as policy choices reflecting different priorities: stricter thresholds favor proximity but may leave peripheral areas underserved, whereas more permissive thresholds improve territorial coverage at the cost of longer acceptable travel times.

The pattern observed in L’Aquila reflects broader European inequalities driven by population sparsity and uneven service distribution. In Finland, “medical-desert” areas affect approximately one in eight residents, while in Spain, hospital accessibility remains markedly lower than primary-care accessibility [8,24,25]. Across these settings, digital integration has proven essential, as provider-to-provider telehealth and networked services enhance diagnostic accuracy, treatment decisions, and follow-up, particularly for time-critical conditions [19,21]. The emerging “Hub and Spoke 2.0” frameworks take it a step further, incorporating telemedicine, cloud-connected devices, and AI-assisted decision support to extend specialist reach and optimize capacity [16,22,42]. For mountainous territories, this means that physical proximity and digital connectivity must evolve in tandem if equity in healthcare access is to be maintained.

From a methodological standpoint, adopting a network-based travel-time core enhances realism and avoids the distortions of simple Euclidean or coarse raster models. Earlier comparisons have shown that methodological choices can determine who is considered underserved [35,36]. Our results align with recent optimization studies, which suggest that well-calibrated layouts can reduce extreme travel times while more evenly distributing demand [8]. When supply–demand balance or facility competition is crucial, floating-catchment and gravity models can complement the analysis. However, they require richer capacity data and remain less intuitive for policy audiences. A travel-time-based approach supported by sensitivity testing, therefore, provides a pragmatic compromise for health-system governance under DM 77/2022.

Several limitations should be considered. The analysis focuses on a single province and therefore reflects its specific geography, demographics, and infrastructure; thresholds for “equitable coverage” may vary in other regions [5,7]. Accessibility estimates are based on modeled road travel times, which may not capture actual mobility behavior in the absence of empirical or multimodal data [29,36,41]. Similarly, because the model relies on estimated rather than observed utilization patterns, it captures potential territorial accessibility rather than actual service uptake or effective demand. The model also does not account for quality of care, clinical outcomes, or patient preferences, and workforce availability was represented only through population caps rather than detailed staffing data, even though recruitment and retention strongly influence accessibility [24,43,44]. Moreover, digital activity was not yet incorporated, despite growing evidence that telehealth can significantly reshape territorial access [19,21,22,45]. Finally, the study provides a cross-sectional snapshot; continuous monitoring would help track seasonal mobility, infrastructure upgrades, and policy reforms over time [8]. Further sensitivity testing of key heuristic assumptions, including alternative spoke-cap thresholds and candidate-margin specifications, would represent a useful next step in future extensions of the framework.

The findings have several implications for health planning and governance. Incorporating measurable accessibility standards, such as the 30 min benchmark, into the monitoring framework of DM 77/2022 would allow planners to assess whether new community health centers and community hospitals truly enhance territorial equity [24,41,46]. In practice, this would require periodic updating of the allocation model using official demographic data, travel-time matrices, and the current configuration of active hub-and-spoke facilities under the coordination of planning, information systems, and district-level functions within the Local Health Authority. In subsequent iterations, the framework could be further refined by incorporating staffing availability, service-activity volumes, and telehealth-supported care pathways. Infrastructure investment should proceed hand in hand with digital development, ensuring that broadband connectivity, interoperable electronic records, and teleconsultation workflows expand alongside the physical network so that distance becomes a manageable parameter rather than a barrier [16,22,42,47]. Workforce equity remains central, as targeted incentives, rotational appointments between hubs and spokes, and the inclusion of community nurses and telehealth coordinators can help reduce professional isolation and sustain peripheral services [24,43,44]. At the same time, governance should embrace geospatial scenario modeling as a standard evaluation tool, using open and auditable methods to anticipate the effects of demographic change, service reconfiguration, and infrastructure investment, in line with broader discussions on digitally enabled and strategically managed public services [7,8,11,33,37].

## 5. Conclusions

This work demonstrates how algorithmic allocation, based on travel time and explicit capacity thresholds, can translate the principles of DM 77/2022 into quantifiable and testable planning targets [37,38,39]. Ensuring universal access is not simply a matter of building more facilities but of optimizing their spatial configuration and linking them digitally and professionally to preserve coherence and sustainability [17,22]. Although the analysis focused on a single Italian province, the methodology can inform national and European strategies to reduce medical deserts and embed equity within territorial networks [16,33]. Beyond its local application, the proposed framework demonstrates national and international scalability, as its data-driven and open-source structure can be adapted to different territorial systems and policy environments to promote equitable healthcare access. By combining geography, policy, and technology, the hub-and-spoke model provides a practical framework for operationalizing spatial justice in everyday healthcare, where proximity, quality, and sustainability reinforce one another.

## Figures and Tables

**Table 1:** Population served and mean travel times per hub-and-spoke (baseline scenario, 30 min threshold).

Area	Center Type	Population Served	Mean Travel Time (min)	Mean Distance (km)	Population Density (inh./km^2^)
AVEZZANO	Hub	48,319	14	13	176.4
Carsoli	Spoke	15,234	15	13	48.4
Civitella Roveto	Spoke	17,814	14	12	44.6
Trasacco	Spoke	31,706	17	16	50.6
L’AQUILA	Hub	69,717	19	16	147.1
Montereale	Spoke	13,101	19	16	34.3
Rocca di Mezzo	Spoke	16,797	19	18	38.9
San Demetrio ne’ Vestini	Spoke	12,362	19	16	20.1
SULMONA	Hub	40,334	19	14	61.8
Castel di Sangro	Spoke	14,349	24	19	28.0
Castelvecchio Subequo	Spoke	7099	15	12	19.4

The table shows the population served, mean travel time, mean distance, and population density for each hub-and-spoke center under the baseline configuration (30 min scenario).

**Table 2:** Scenario analysis under alternative travel-time thresholds.

Scenario (Cut-Off)	Municipalities Allocated	Population Allocated (%)	Mean Travel Time (min)	90th Percentile (min)	Maximum Travel Time (min)	Constraint Violations
20 min	54	88	14	19	22	11 mountainous municipalities excluded
30 min (baseline)	65	100	17	28	32	L’Aquila Hub > 50,000 residents
30 min (cap-compliant)	65	100	18	34	38	None
40 min	65	100	20	29	36	None

The table reports allocation feasibility, travel-time distribution, and capacity-constraint violations under 20, 30, and 40 min planning benchmarks. “Allocated” indicates municipalities/populations that could be assigned under the scenario assumptions.

## Data Availability

The data used in this study are derived from official datasets provided by the Italian National Institute of Statistics (ISTAT), including municipal demographic data and the national origin–destination road travel-time matrix (reference year: 2021). These datasets are publicly available from ISTAT institutional repositories. Processed datasets and the allocation algorithm outputs are available from the corresponding author upon reasonable request, in accordance with data-sharing policies.

## Supplementary Materials

The following supporting information can be downloaded at: https://www.mdpi.com/article/10.3390/healthcare14070915/s1, Algorithm S1. Heuristic Allocation Procedure for Hub-and-Spoke Assignment. Figure S1. Geographic location of the Province of L’Aquila within Italy. The highlighted area identifies the ASL 1 Avezzano-Sulmona-L’Aquila study area within the Abruzzo Region. Figure S2. Municipality-level assignments, travel times, and distances in the ASL 1 hub-and-spoke network. Panels show detailed cartographic outputs for the L’Aquila area (A), Marsica area (B), and Peligno-Sangrina area (C). For each municipality, travel time (minutes) and distance (km) to the assigned hub or spoke node are reported.

## Abbreviations

ASL: Local Health Authority (Azienda Sanitaria Locale); DM: Ministerial Decree; ISTAT: Italian National Institute of Statistics; OD: origin–destination; LEA: Essential Levels of Care; PNRR: National Recovery and Resilience Plan.
