# Supplementary material for: Optimizing Territorial Healthcare Networks with a Capacity-Constrained Hub-And-Spoke Allocation Algorithm: The Province of L’Aquila Case Study
Source: Healthcare (Basel). 2026 Apr 1;14(7):915. doi: 10.3390/healthcare14070915 (PMC13073968; doi:10.3390/healthcare14070915)
Supplement: Supplementary file 1 [file healthcare-14-00915-s001.zip › healthcare-4173373-supplementary.pdf]

Algorithm S1. Heuristic Allocation Procedure for Hub-and-Spoke Assignment

Input: municipalities in the Province of L'Aquila; candidate hub and spoke nodes; origin-destination travel-time matrix; municipal populations; hub and spoke population caps; scenario-specific travel-time threshold.

Output: baseline proximity-first allocation; cap-compliant allocation; unassigned municipalities, if any.

1. Compute travel time from each municipality to all candidate nodes.
2. Exclude candidate nodes whose travel time exceeds the scenario-specific threshold.
3. If no feasible candidate node remains after threshold filtering, mark the municipality as unassigned.
4. For each municipality with at least one feasible candidate, identify the minimum travel time.
5. Define the near-optimal candidate set as all candidate nodes within 5% of the municipality-specific minimum travel time.
6. Sort municipalities by:
   (a) ascending number of feasible candidate nodes;
   (b) ascending minimum travel time;
   (c) descending population size.
7. If ties remain, apply a fixed ordering rule.
8. Tentatively assign each municipality to its preferred feasible candidate within the near-optimal set.
9. If the preferred assignment exceeds the population cap, activate iterative reallocation.
10. During reallocation, move selected municipalities to the next-best feasible alternative in order to reduce overload while minimizing additional travel time.
11. Continue the reallocation cycle until capacity violations are resolved or no further feasible reassignment can be identified.
12. Record both the baseline proximity-first allocation and the final cap-compliant allocation.
13. Export final assignment tables and summary indicators.

Reproducibility: under identical inputs, thresholds, and ordering rules, the procedure yields the same allocation output.

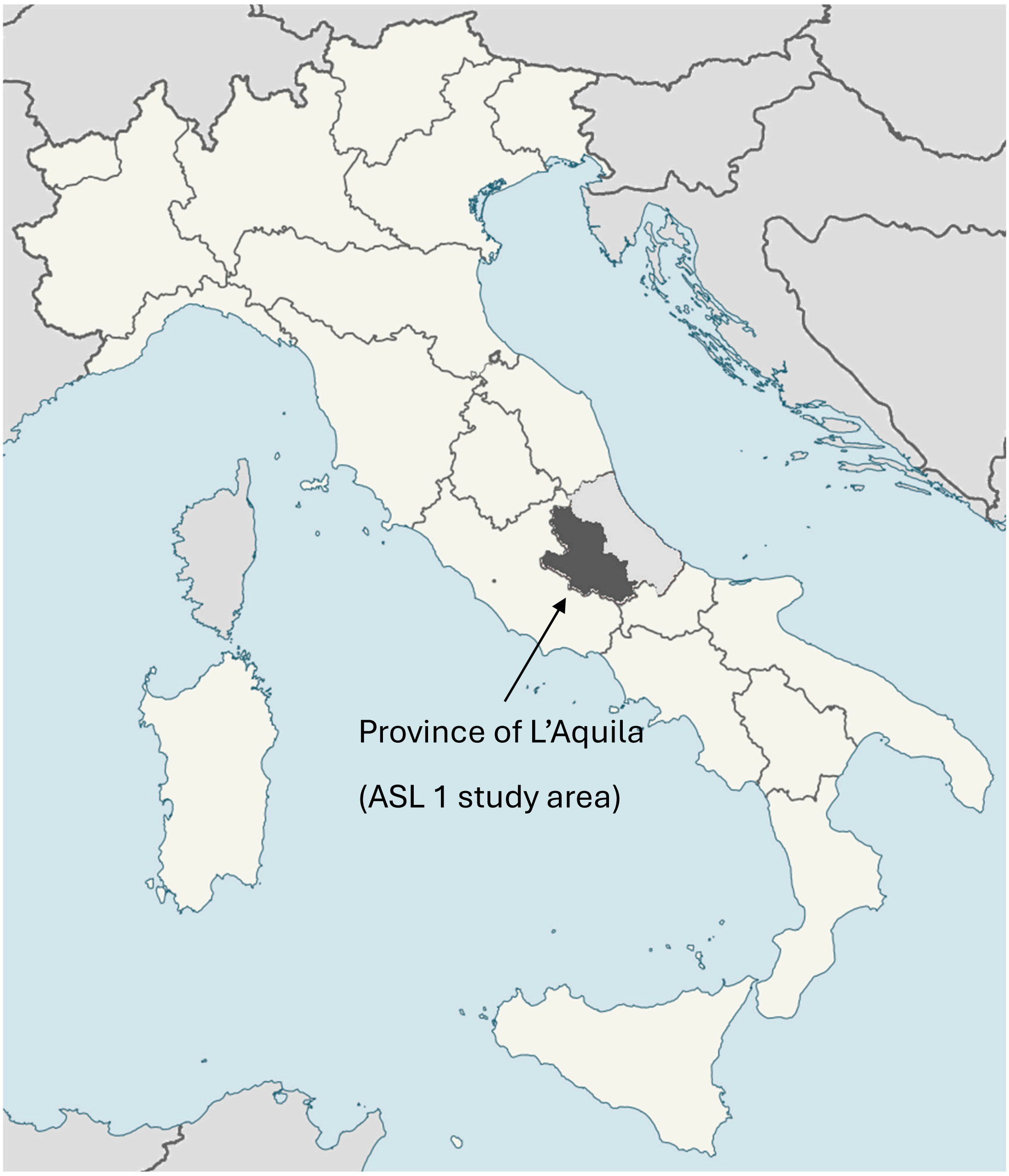

Supplementary Figure S1. Geographic location of the Province of L'Aquila within Italy. The highlighted area identifies the ASL 1 Avezzano-Sulmona-L'Aquila study area within the Abruzzo Region.

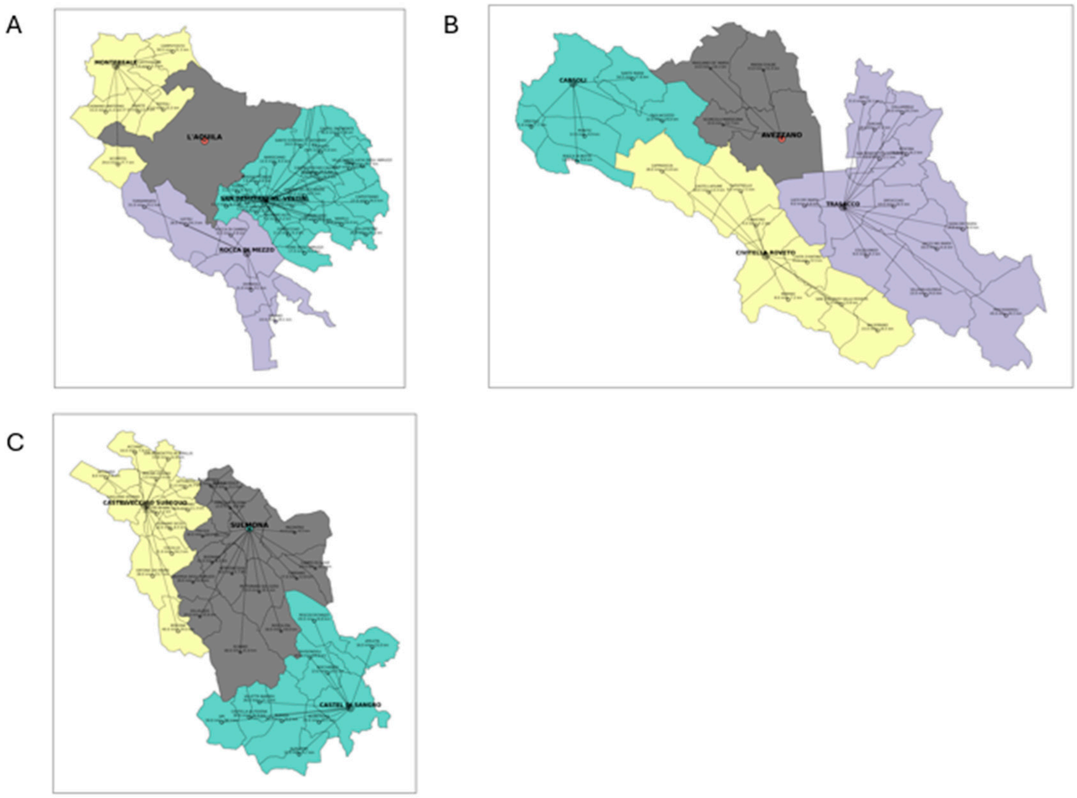

Supplementary Figure S2. Municipality-level assignments, travel times, and distances in the ASL 1 hub-and-spoke network. Panels show detailed cartographic outputs for the L'Aquila area (A), Marsica area (B), and Peligno-Sangrina area (C). For each municipality, travel time (minutes) and distance (km) to the assigned hub or spoke node are reported
